# Dichloroacetate Decreases Cell Health and Activates Oxidative Stress Defense Pathways in Rat Alveolar Type II Pneumocytes

**DOI:** 10.1155/2015/129031

**Published:** 2015-08-02

**Authors:** Alexis Valauri-Orton, Frizzi Bschorer, Karen K. Bernd

**Affiliations:** Biology Department, Davidson College, Davidson, NC 28035, USA

## Abstract

Dichloroacetate (DCA) is a water purification byproduct that is known to be hepatotoxic and hepatocarcinogenic and to induce peripheral neuropathy and damage macrophages. This study characterizes the effects of the haloacetate on lung cells by exposing rat alveolar type II (L2) cells to 0–24 mM DCA for 6–24 hours. Increasing DCA concentration and the combination of increasing DCA concentration plus longer exposures decrease measures of cellular health. Length of exposure has no effect on oxidative stress biomarkers, glutathione, SOD, or CAT. Increasing DCA concentration alone does not affect total glutathione or its redox ratio but does increase activity in the SOD/CAT oxidative stress defense pathway. These data suggest that alveolar type II cells rely on SOD and CAT more than glutathione to combat DCA-induced stress.

## 1. Introduction

Individuals may be exposed to haloacetic acids, and specifically dichloroacetate (DCA), via multiple sources. A byproduct of the chlorine disinfection process used in municipal water systems and the* in vivo *breakdown of the industrial solvent trichloroethylene, DCA is one of the most prevalent tap water contaminants in America with drinking water levels including up to 133 *μ*g/L DCA [1–3]. Due to its environmental presence, individuals are directly impacted by DCA through inhalation of aerosols, such as in showers and chlorinated pools [3, 4], and through the blood stream after ingestion [3]. For some individuals these environmental exposures are augmented by therapeutic doses of 10–50 mg/kg/day, as there are promising clinical applications for DCA in treatment of metabolic dysfunctions and antitumor activity [3, 5–14]. Keys to developing DCA's medicinal potential and properly regulating its environmental impacts lie in understanding DCA's toxicity in different cell types.

DCA's cellular impact is dependent upon duration of exposure and genetic background. Glutathione transferase 1 (GSTz1) converts DCA to the nontoxic metabolite, glyoxylate. However, GSTz1 is polymorphic with alleles showing variable inhibition due to repeated DCA exposure. As a result, in a subset of individuals therapeutic doses of DCA would induce DCA accumulation to potentially toxic levels [15–20]. Environmental exposure to DCA, while at significantly lower than therapeutic levels, is likely to be chronic and, therefore, could manifest increased damage due to repeated exposure over time.

Because of the clinical potential and environmental presence of this compound it is important to characterize DCA's effects on cells where accumulation is likely to be the highest. Previous research shows that DCA exposure at and above clinical concentrations induces oxidative stress biomarkers and decreases viability in hepatocytes, is hepatocarcinogenic, and leads to peripheral neuropathy [21–27]. The liver, neurons, and astrocytes, however, are exposed to DCA only through circulation. In contrast, lung cells have two exposure routes, circulation, and inhalation, which could lead to increased effective doses in this tissue.

Lung alveoli walls contain type I and type II pneumocytes and macrophages. While DCA's effect on pneumocytes has not been characterized, J774.1 macrophage cells exposed to DCA revealed time-dependent decreases in viability and in the antioxidant, glutathione, and time-dependent increases in superoxide dismutase (SOD) activity [28–30]. Until this time, however, the effect of DCA on type I or type II pneumocytes has not been reported. Type II cells secrete pulmonary surfactant, and, in response to lung damage, proliferate and differentiate into the type I pneumocytes that are responsible for gas exchange [31, 32]. Damage to type II pneumocytes is highly detrimental to lung function and, thus, understanding DCA's effects in type II pneumocytes is critical to the evaluation of its toxicological impact. To allow direct comparison between cell types, this study's design is modeled after previous work using 0–50 mM exposure to investigate peripheral neuropathy and liver and macrophage cell function [28–30, 33]. It extends our knowledge to include the effect of DCA on lung cells, using rat alveolar type II (L2) cells as a model.

## 2. Materials and Methods

### 2.1. Chemicals

Fetal bovine serum (FBS) was obtained from Atlanta Biologicals (Lawrenceville GA). Phenol-red-free, low glucose DMEM, and dichloroacetate (DCA) were purchased from Sigma-Aldrich (St. Louis, MO). 100x antibiotic-antimycotic was purchased from Gibco (Carlsbad, CA). Trypsin-EDTA 0.05% was obtained from VWR (Radnor, PA).

### 2.2. Cell Culture

Female rat noncancerous type II alveolar cells (L2, cc-149) were obtained from ATCC. L2 stocks were maintained in low glucose DMEM/10% FBS/1x antibiotic, antimycotic at 37°C + 6% CO_2_. Cells were fed every 3–5 days and split when they reached 80–100% coverage. Cells were split via trypsinization, seeded into the tissue culture treated vessel indicated for that analysis, and prior to experimental treatments were allowed 16 hr to acclimate and attach. Replicates exposed to 0 mM, 8 mM, 16 mM, and 24 mM of DCA for 0, 6, 16, or 24 hours were used for analysis.

### 2.3. Determination of Cell Viability

Cells were seeded into clear bottom 96-well plates at 10^5^ cells/well (Corning, Corning, NY) and allowed to attach before the indicated DCA treatment. To determine relative viability, mitochondrial activity was assayed as per manufacturer's instructions (MTT Kit, Roche, Indianapolis, IN). The Abs_655_ (background) and Abs_600_ (formazan) were measured via Model 680 Microplate Reader (Bio-Rad). Prior to statistical analysis, values in each exposure time group were normalized to the control average (0 mM DCA) for that exposure time to control for slight variations in number amongst cells seeded *n* = 6.

### 2.4. Lysate Preparation for Antioxidant Enzyme Studies

Cells were seeded into 10 cm^2^ tissue culture flask-tubes (TPP-US, St. Louis, MO) at 10^6^ cells per tube and treated with DCA as indicated in the text. Cells were harvested via scraping and centrifugation (2,000 g × 10 min, 4°C). Growth media were removed and cells resuspended in 1 mL of 50 mM potassium phosphate (pH 7.0) + 1 mM EDTA. Lysates were prepared via cycling three times between LiqN_2_ and 37°C. Lysates were cleared via centrifugation (2,000 g × 5 minutes) and the supernatants collected and stored at −80°C. Assays were performed within 2 weeks of lysate preparation.

### 2.5. Determination of Superoxide Dismutase and Catalase Activity

Catalase (EC 1.11.1.6) and SOD (EC 1.15.1.1) activity were assayed from the same lysate sample and determined as per manufacturer's instructions (catalase kit and SOD kit, Cayman Chemical Company, Ann Arbor, MI). Absorbance values at 540 nm (catalase assay) and 450 nm (SOD assay) were determined via Model 680 Microplate Reader (Bio-Rad). For each time and concentration combination SOD *n* = 10–12 and catalase *n* = 5-6.

### 2.6. GSH/GSSG-Glo Assay

Cells were seeded in white bottom 96-well plates at 10^4^ cells/well (Corning, Corning, NY), allowed to attach, and treated with DCA. Prior to the assay growth media were removed and cells washed with PBS. Total glutathione and GSSG were each assayed in triplicate via GSH/GSSG Glo kit (Promega, Madison, WI) following manufacturers instructions and as in Chalfant and Bernd [34].

### 2.7. Statistical Analysis

Data obtained from independently prepared cultures were analyzed using Microsoft Excel and JMP software. Values from each exposure time sample were normalized to the mean 0 mM DCA samples value for that exposure time. Outliers were identified and removed using a* Q*-test with a 90% confidence interval. Data presented are the normalized mean ± SD. To determine the effects of independent variables DCA concentration and exposure length on the different biological markers, two-way ANOVA tests were performed followed by LSMeans Differences Tukey HSD* post hoc* test (*p* < 0.05).

## 3. Results

**Figure 1:**
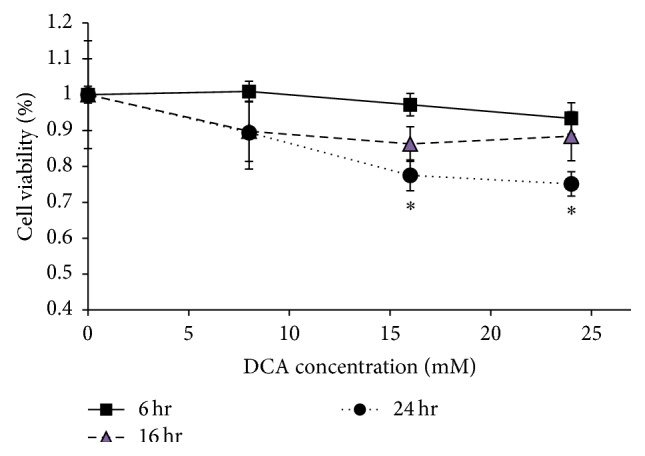
Effect of DCA treatment regimes on L2 viability. Cells were exposed to 0, 8, 16, and 24 mM DCA for 6, 16, and 24 hours. Viability was determined by MTT assay with the viability 0 mM DCA treatment for the corresponding exposure time defined as 100%. Bars represent mean ± S.D., *n* = 5-6. *∗* denotes a significant difference between data from that condition and those without *∗* (*p* < 0.037).

To investigate the effects of DCA on rat alveolar type II (L2) cells, samples were treated with 0–24 mM DCA at the indicated time points and the MTT assay was used to determine percent viability. Both the duration of DCA exposure and the concentration of DCA in the treatment resulted in significant decreases in cell viability (Figure 1). While no length of exposure to 8 mM DCA compromised cell health, exposure to 16 mM or 24 mM DCA for 24 hours induced significant decreases in viability, 22 and 25%, respectively. In addition, statistical analysis revealed a significant interaction effect between the two variables, indicating that the combination of increased duration of exposure and higher DCA concentrations causes a more severe decline in cell health than changes in either variable alone (*p* < 0.037).

**Figure 2:**
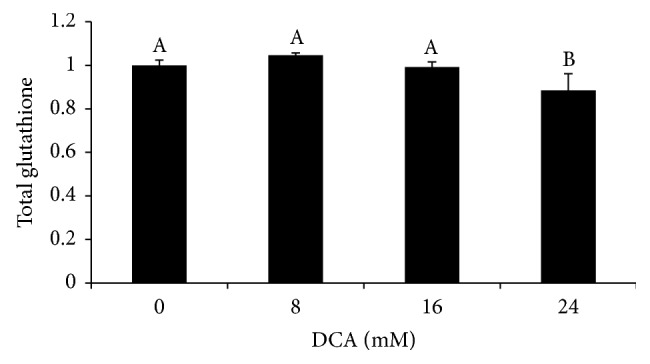
Effect of DCA on total glutathione. Cells were exposed to 0, 8, 16, and 24 mM DCA. Measurements of total glutathione at 8, 16, and 24 hr DCA exposure were pooled and that average value normalized to baseline glutathione levels (0 mM DCA) and the mean ± S.D. is shown. *n* = 9. Bars with no shared superscripts are significantly different (*p* < 0.0008).

Because DCA significantly reduced cellular viability and the compound is known to induce oxidative stress in other cell types, the roles of different antioxidant pathways were characterized. Glutathione, a general antioxidant, exists in reduced and oxidized pools within the cytoplasm and mitochondria. A cell's exposure to oxidant can cause changes in the total amount of glutathione or may shift the ratio of glutathione in reduced versus oxidized forms. To examine the effect of DCA exposure, total glutathione in samples treated with the indicated concentration of DCA for 8, 16, or 24 hr was pooled and that average normalized to the 0 mM treatment. These data reveal a downward trend where increases in DCA concentration to 24 mM have a significant negative effect on total glutathione levels (Figure 2; *p* < 0.0008). The role of glutathione was further characterized by determining the effect of DCA on the antioxidant's oxidation state. Analysis of the amount of oxidized glutathione (GSSG) and the ratio of reduced to oxidized glutathione (GSH: GSSG) revealed that neither DCA concentration nor exposure time had a significant effect on levels of oxidized glutathione or on the ratio of reduced/oxidized glutathione (data not shown).

**Figure 3:**
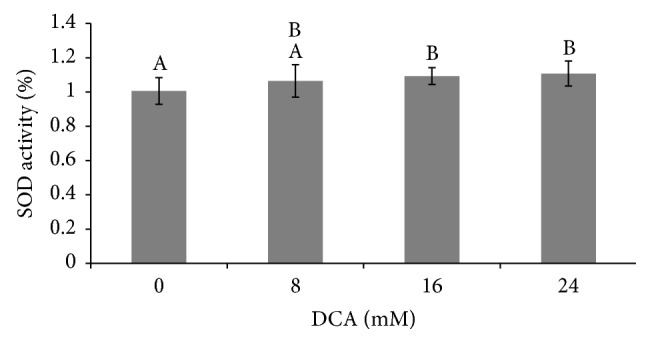
Effect of DCA on L2 cell SOD activity. Cells were exposed to DCA at concentrations of 0, 8, 16, and 24 mM. SOD activity at 8, 16, and 24 hr DCA exposure was pooled and that average value normalized to baseline SOD levels (0 mM DCA) and mean ± S.D. is shown. *n* = 33–36. Columns with no shared superscripts are significantly different (*p* < 0.003).

Superoxide dismutase (SOD) and catalase (CAT) function in an antioxidant pathway that detoxifies superoxide anion to molecular oxygen and water. The duration of exposure to DCA (0–24 hr) did not have an effect on this metric and no duration: concentration interaction effect was seen. However, SOD activity increases as the DCA treatment concentration rises (Figure 3). Treatment of L2 cells with DCA concentrations of 16 mM and 24 mM resulted in the increase of SOD activity by 9 and 11%, respectively, over the corresponding control (*p* < 0.0003).

**Figure 4:**
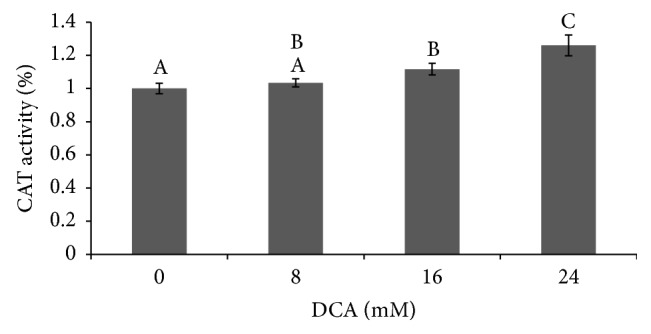
Effect of DCA on L2 cell CAT activity. Cells were exposed to DCA at concentrations of 0, 8, 16, and 24 mM. CAT activity at 8, 16, and 24 hr DCA exposure was pooled and that average value normalized to baseline CAT levels (0 mM DCA) and mean ± S.D. is shown. *n* = 20–24. Columns with no shared superscripts are significantly different (*p* < 0.0001).

CAT activity changes in response to DCA treatment are similar to those seen for SOD activity. Again, length of exposure did not affect the level of CAT activity and no significant interaction effect was seen between concentration and length of exposure. However, DCA concentration alone has a significant effect on CAT activity with 16 mM and 24 mM DCA treatments significantly increasing CAT activity (12 and 26%) as compared to the control treatments (Figure 4, *p* < 0.0001).

## 4. Discussion

Previous studies have found DCA to be hepatotoxic, hepatocarcinogenic, damaging to macrophage cells, and capable of inducing peripheral neuropathy* in vivo* [21–31, 33]. This study extends our understanding to a novel cell type, lung cells, and illustrates that DCA has a significant, negative effect on rat alveolar type II (L2) cells.

L2 cells show a distinct pattern of DCA sensitivity. Both the level and the duration of DCA exposure decrease L2 cell viability, with statistical analysis revealing a significant cross effect between the variables. This places L2 cell's 16 mM DCA threshold sensitivity between those reported for CHO cultures (2.0 mM DCA [35]) and J774A.1 macrophages (24 mM [31]).

Glutathione is known to be a component of lung, and particularly alveolar, antioxidant defense system [14, 36, 37]. Macrophages challenged with 24 mM DCA for 24–60 hr demonstrate time-dependent decreases in total glutathione [31]. By comparison, 0–24 hr DCA exposures caused no changes in L2 cell's total glutathione levels. Initially, statistical analysis revealed a significant glutathione decrease in L2 cells exposed to 16 mM or 24 mM DCA. However, the statistical correlation between decreases in cell viability and total glutathione accounts for 73% of the decrease in the antioxidant seen at exposures ≥8 mM. The small difference that remains is not significant. Therefore, while 24 mM DCA affects glutathione levels in macrophages, the conditions tested do not alter glutathione levels in a second component of alveoli, L2 cells.

Because DCA-induced decreases in glutathione have been reported in other culture and whole animal systems [23, 24, 29–31], the lack of a significant effect on total glutathione levels in L2 cells was unexpected and investigated further. In addition to altering total glutathione levels, oxidative stress can shift the GSH: GSSG ratio [38]. However, neither DCA concentration nor exposure length caused significant changes in oxidized glutathione levels or in the GSH: GSSG ratio. These data suggest that glutathione is not a major component of type II alveolar cell response to oxidative stress caused by acute DCA exposure.

DCA-induced superoxide anion production is expected to activate the SOD/CAT oxidative stress pathway. Consistent with our findings on DCA toxicity, L2 cells demonstrate a different and more sensitive SOD/CAT response than macrophage cells [28, 31]. In L2 cells, increases in SOD/CAT activity are dependent upon DCA concentration but not upon exposure duration. Treatment with 16 mM DCA causes similar increases in SOD and CAT activity (9% versus 12%). However, 24 mM DCA results in greater induction of CAT than SOD (26% versus 11%). The increases in catalase activity seen after exposure to DCA are consistent with the cell responding to H_2_O_2_ produced by SOD. However, in L2 cells, DCA's induction of CAT activity surpasses its induction of SOD activity. Reports show that DCA induces H_2_O_2_ production by hepatocyte peroxisomes [34]. Therefore the higher induction of CAT activity seen here could be due to 24 mM DCA exposure triggering similar peroxisome activity increases in L2 cells. However, the differences previously noted between CHO cells, macrophages, and L2 cells indicate that the complexity of the system supports future empirical testing of whether the catalase activity induction seen in L2 cells is due to peroxisome activity or a byproduct of increased SOD activity.

In conclusion, previous research has shown DCA has deleterious effects on liver and neuronal cells but these organs only experience the compound through the blood stream. Because of their position at the interface between the internal and external environments, lung compartments experience exposure to this industrial solvent breakdown product/water purification byproduct through the blood stream and via inhalation. Lung alveoli are at this junction point in the body's defenses against oxidative damage. Previously only their macrophage component of lungs had been examined. We show that type II cells are more DCA-sensitive than macrophages and respond by differential activation of pathways that detoxify superoxide anion and H_2_O_2_. Our results further highlight the need to investigate DCA's effects in multiple systems, as threshold sensitivity levels and responses vary among cell types. The fact that type II pneumocytes are negatively affected by exposure to lower concentrations of environmental DCA underscores the importance of multisystem studies when establishing exposure limits.
